# HTLV-1 Rex Tunes the Cellular Environment Favorable for Viral Replication

**DOI:** 10.3390/v8030058

**Published:** 2016-02-24

**Authors:** Kazumi Nakano, Toshiki Watanabe

**Affiliations:** Department of Computational Biology and Medical Sciences, Graduate School of Frontier Sciences, The University of Tokyo, 4-6-1, Shirokanedai, Minatoku, Tokyo 108-8639, Japan; tnabe@ims.u-tokyo.ac.jp

**Keywords:** HTLV-1 Rex, pro-viral expression, unspliced RNA, NMD, alternative splicing, cell cycle regulation

## Abstract

Human T-cell leukemia virus type-1 (HTLV-1) Rex is a viral RNA binding protein. The most important and well-known function of Rex is stabilizing and exporting viral mRNAs from the nucleus, particularly for unspliced/partially-spliced mRNAs encoding the structural proteins essential for viral replication. Without Rex, these unspliced viral mRNAs would otherwise be completely spliced. Therefore, Rex is vital for the translation of structural proteins and the stabilization of viral genomic RNA and, thus, for viral replication. Rex schedules the period of extensive viral replication and suppression to enter latency. Although the importance of Rex in the viral life-cycle is well understood, the underlying molecular mechanism of how Rex achieves its function has not been clarified. For example, how does Rex protect unspliced/partially-spliced viral mRNAs from the host cellular splicing machinery? How does Rex protect viral mRNAs, antigenic to eukaryotic cells, from cellular mRNA surveillance mechanisms? Here we will discuss these mechanisms, which explain the function of Rex as an organizer of HTLV-1 expression based on previously and recently discovered aspects of Rex. We also focus on the potential influence of Rex on the homeostasis of the infected cell and how it can exert its function.

## 1. Molecular Events in the Host Cell Caused by HTLV-1 Infection

Infection of T-cells with human T-cell leukemia virus type 1 (HTLV-1) causes adult T-cell leukemia (ATL), HTLV-I associated myelopathy/tropical spastic paraparesis (HAM/TSP) and HTLV-1 uveitis (HU) [1,2], although the molecular basis of such variations in the pathogenesis of HTLV-1 has not been fully elucidated. The structure of the genomic HTLV-1 RNA and the molecular events triggered by HTLV-1 infection have been thoroughly investigated [3,4,5,6,7]. Briefly, the genomic RNA of HTLV-1 is composed of 8685 nucleotides with two long terminal repeats (LTRs), which function as the viral promoter, at the both 5′ and 3′ ends. Although the genomic RNA is compact, HTLV-1 has various RNA signals to obtain the most out of its coding potential. By utilizing (1) three overlapped reading frames with two -1 programmed ribosomal frameshift signal (-1PRF), (2) two alternative splicing sites, (3) and multiple start and stop codons, HTLV-1 genomic RNA encodes more than 10 viral proteins [8]. HTLV-1 has three alternatively-spliced forms of viral mRNAs, which are unspliced, singly (partially)-spliced and doubly (fully)-spliced. The unspliced HTLV-1 mRNA encodes Gag, Pro, and Pol proteins, while singly-spliced RNA encodes Env. The doubly-spliced HTLV-1 mRNA encodes functional accessory proteins, such as Tax, Rex, P30II, p12, p13 in sense open reading frames (ORFs) and HBZ (HTLV-1 basic leucine zipper factor protein) in an anti-sense ORF.

**Figure 1:**
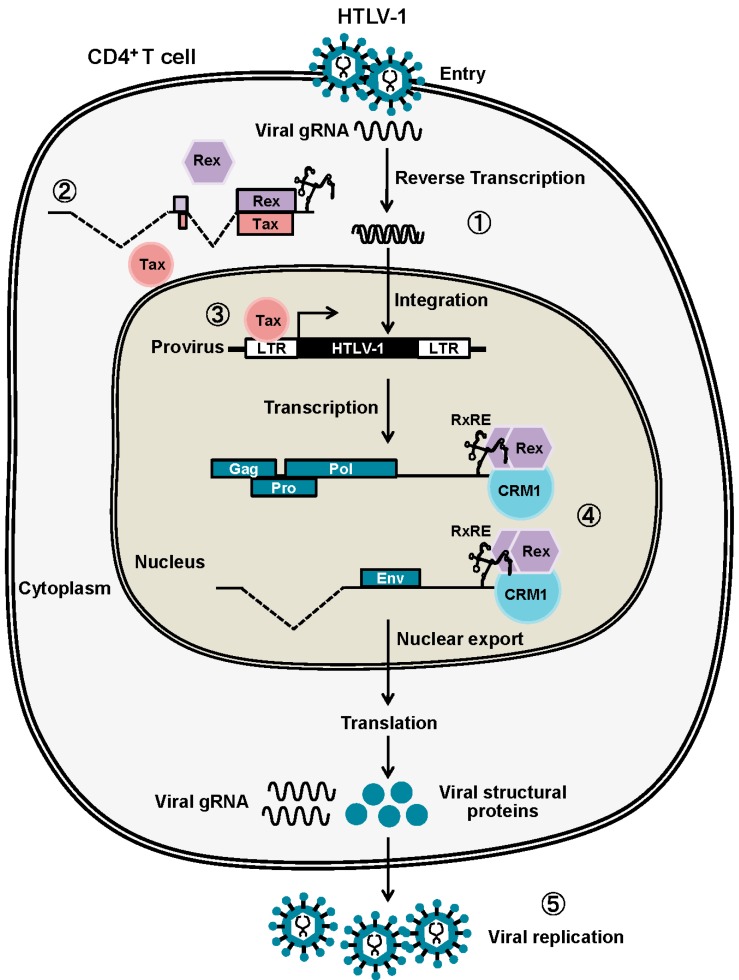
After HTLV-1 entry, the viral genomic RNA is reverse-transcribed and integrated into the host human genome (1). The viral mRNA from the provirus for the first-round of transcription is completely spliced to *tax/rex* mRNA by the cellular splicing machinery (2). Tax stimulates the transactivation of LTRs for further viral transcription, resulting in the gradual accumulation of Rex in the infected cell (3). Rex then starts exporting the unspliced and partially spliced viral mRNAs, encoding Gag, Pro, Pol, and Env, to the cytoplasm by binding to RxRE of viral mRNA (4),resulting in active viral replication (5). Due to active nuclear export of unspliced and partially spliced viral mRNA by Rex, that of *tax/rex* mRNA is eventually reduced to enter the latency.

After integration to the human genome, transcription and translation from the HTLV-1 provirus rely entirely on the host cell machinery. The translated viral accessory proteins then function in a precise schedule for effective viral replication [9,10] (Figure 1). The viral mRNA from the provirus for the first round of transcription is completely spliced to *tax/rex* mRNA by the cellular splicing machinery. Tax is more effectively translated from *tax/rex* mRNA because of its stronger Kozak sequence compared with that of Rex [11]. Then, Tax stimulates the transactivation of LTRs for enhanced *tax/rex* mRNA transcription. Such feed-forward activation of the HTLV-1 provirus results in the gradual accumulation of Rex in the infected cell. Subsequently, accumulation of sufficient Rex permits Rex-mediated nuclear export of unspliced and partially spliced viral RNA. The active export of these viral mRNAs to the cytoplasm by Rex results in enhanced translation of the viral structural proteins, Gag, Pro, Pol, and Env and, thereby, enhances viral replication. Inversely with the active nuclear-export of unspliced and partially-spliced viral mRNA by Rex, that of *tax/rex* mRNA is reduced; thus, cellular concentrations of Tax and Rex proteins are also decreased. Moreover, p30II from the minor doubly-spliced viral mRNA binds and retains *tax/rex* mRNA in the nucleoli by its strong nucleolar localization signal (NoLS). In combination, the cellular levels and activities of Tax and Rex proteins are gradually reduced, and both viral expression and replication are diminished to enter the latency. Rende *et al.* [12] mathematically analyzed the molecular events in early-phase HTLV-1 infection and confirmed that viral expression was indeed divided into two phases. The first phase was Tax/Rex expression, and the second phase was structural protein expression, which were both controlled by the functions of Tax and Rex. Furthermore, they concluded that the two-phase kinetics of HTLV-1 expression was strictly regulated by Rex, indicating that Rex is the major conductor of HTLV-1 expression.

## 2. Canonical Rex Function as a Post-Transcriptional Regulator of Viral Expression

### 2.1. Rex-Dependent Nuclear Export of Viral mRNAs

Rex binds to the Rex Responsive Element (RxRE) of the HTLV-1 mRNAs to form Rex-viral mRNA complex for selective nuclear-export. Unlike the Rev Responsive Element (RRE) in human immunodeficiency virus type-1 (HIV-1) mRNAs, RxRE is in all HTLV-1 mRNAs [5,13]. The RxRE of HTLV-1 mRNA maps to the region of 255 nucleotides (nt) from the U3 to the R region of the 3′-LTR and forms a stable secondary structure with four stem loops. Such a unique structure of RxRE is considered to function as the landmark for Rex to selectively bind to the viral mRNAs [14,15]. Although, all HTLV-1 derived mRNAs have RxRE, the nuclear export efficiency by Rex is different among HTLV-1 mRNAs. It has been widely accepted that cytoplasmic accumulations of unspliced and partially-spliced HTLV-1 mRNAs are Rex dependent, while that of fully spliced *tax/rex* mRNA is suppressed by Rex [12,16]. Subsequently, Bai *et al.* demonstrated that Rex also stimulated the nuclear-export of *tax/rex* mRNA and Tax expression, at least partially [17]. Most recently, Cavallari *et al.* elegantly demonstrated that not only unspliced *gag/pol* mRNA and singly spliced *env* mRNA, but also some of singly- and fully-spliced mRNAs encoding viral accessory proteins were also nuclear-exported in Rex-dependent manner. They showed that *p30II*, *p12/p8*, and *p13* mRNAs were Rex-dependent, while *tax/rex* and *p21rex* mRNAs were Rex-independent. Interestingly, all Rex-dependent viral mRNAs contain 75 nt intronic regions, which control Rex-dependency as a *cis*-acting sequence [18]*.* Another study group demonstrated that HBZ, the antisense protein of HTLV-1, inhibited the nuclear-export of intron-containing mRNA by Rex, thus inhibited active viral replication and induced latency [19]. These reports suggest that Rex-mediated nuclear exports of HTLV-1 mRNAs are finely-tuned by RxRE, inherent *cis*-acting viral sequence, and viral proteins.

Rex binds to Chromosomal Maintenance 1 **(**CRM1), also known as Exportin 1 (XPO1), via its nuclear export signal (NES) for nuclear export. CRM1 is a cellular nuclear export protein which is responsible for the translocation of various cellular proteins with NES. Thus, the export of HTLV-1 mRNAs to the cytoplasm is dependent on CRM1, which is separated from the bulk cellular mRNAs exported in an Aly/Ref export-factor-dependent manner. The molecular mechanism of the RxRE-Rex-CRM1 complex formation has been extensively studied by Hakata *et al.* [20,21]. The authors revealed that the Rex has to be multimerized to bind to RxRE. They also propose a possibility that CRM1 is involved not only in the translocation of Rex but also in its multimerization. Therefore, one may speculate that Rex initially forms a complex with CRM1, which assists oligomerization of Rex on CRM1 before binding to RxRE. Nevertheless, the detailed order of the complex formation has never been investigated.

### 2.2. Primary Structure of Rex and Its Function

HTLV-1 Rex protein consists of 189 amino acids with its molecular weight of approximately 27 kDa. The HTLV-1 Rex protein contains several functional domains essential for its function. The primary structure of Rex has been well-described [22,23]. The N-terminal arginine-rich RNA-binding domain (aa 1–19) is required for binding to RxRE. Additionally, this domain overlaps with the nuclear localization signal (NLS), which is essential for Rex to shuttle-back to nucleus with importin-β, and with the p30II-binding domain. Rex interacts with CRM1 through the NES (aa 66–118) for nuclear export. Rex has two multimerization domains (aa 57–66 and 106–124) with NES in between and both of them are considered to be necessary for stable oligomerization of Rex. Most recently, a stability domain was identified at the C-terminal region of Rex (aa 170–189) [24,25,26]. The authors demonstrated that deletion of the stability domain destabilized Rex significantly but did not influence the function of Rex.

### 2.3. Rex Activity and Phosphorylation

It has been described that the activity Rex is finely regulated through its phosphorylation [27] at several serine(Ser)/threonine(Thr) residues [24]. The treatment of HUT102 (an HTLV-1-infected cell line) with a protein kinase C inhibitor, H-7 [1-(5-isoquinolinyl-sulfonyl)-2-methylpiperazine] destabilized unspliced viral mRNA and reduced the expression level of Gag-p19 protein [27]. To date, seven phosphorylation sites of Rex have been identified at Thr-22, Ser-36, Thr-37, Ser-70, Ser-97, Ser-106, and Thr-174 [24,28]. Kesic *et al.* [24] evaluated the importance of phosphorylation sites and demonstrated that Rex phosphorylation at Ser-97 and Thr-174 was the most critical for the efficiency of RxRE-dependent nuclear export by Rex.

### 2.4. Regulation of Rex by Other HTLV-1 Viral Proteins

As described above, Rex plays a central role in selective expression of HTLV-1 viral structural proteins and is, thus, in active viral reproduction. The Rex activity is critical to switch from the early productive period to late latent period. Therefore Rex activity has to be finely tuned during HTLV-1 infection. HTLV-1 has an elegant auto-regulatory mechanism to regulate the activity of Rex, *i.e.*, by two-phased HTLV-1 expression kinetics, which is described above (see Section 1), and by the function of other viral proteins.

The suppressive function of p30II for the Rex has been well investigated, following extensive reproduction of HTLV-1 virus by Tax and Rex, p30II is expressed from the minor doubly-spliced HTLV-1 mRNA. P30II selectively binds to *tax/rex* mRNA. Then, p30II, with a strong nucleolar localization signal (NoLS), localizes and retained *tax/rex* mRNA in nucleoli, thus preventing their expression and functions. Such time-lagged operations of the positive (Tax and Rex) and negative (p30II) regulators of HTLV-1 promotes the early infectious phase followed by the late infectious phase with a rapid shutdown to escape from the host immune surveillance against pathogens [29,30,31,32]. On the other hand, Rex binds to p30II and rescues *tax/rex* mRNA [32]. The timing of Rex-p30II interaction is considered to regulate switching from the early active-viral-reproduction phase to the late rapid-shutdown phase to escape from the host immune system. Most recently, HBZ, the antisense protein of HTLV-1, was demonstrated to inhibit the nuclear-export of intron-containing mRNA by Rex, thus inhibiting active viral replication and induced latency [19].

Since p21Rex is constitutively expressed in primary peripheral blood mononuclear cells from HTLV-1 carriers and ATL patients [33,34,35], it has been expected that p21Rex plays a role in the HTLV-1 life cycle, such as p27Rex suppressor as a dominant-negative isoform, although, a clear biological function of p21Rex has not been elucidated, yet. P21Rex is expressed from a defective HTLV-1 mRNA without the exon 2, and lacks the N-terminus 78 amino acids of p27Rex, ranging from NLS to the N′-multimerization domain [36,37]. Without NLS, p21Rex localizes to the cytoplasm; thus, the functional importance of this isoform has not yet been elucidated. More recently, Bai *et al.* demonstrated that p21Rex neither nuclear-exported the viral mRNA, nor influenced the p27Rex function [17]. Therefore, p21Rex seems not to be involved in HTLV-1 lifecycle as an isoform of Rex, although possible roles of this short Rex isoform in the cellular biological pathways are required to be elucidated in the future.

## 3. Non-Canonical Functions of Rex: Exploring New Aspects of Rex

### 3.1. NMD Inhibition by Rex

#### 3.1.1. Rex Stabilizes HTLV-1 Genomic RNA by Inhibition of NMD

For viruses, stabilization of viral mRNAs in the host cells is a major issue to overcome for self-replication [38]. Recently, a lot of attention has been directed towards one of the host mRNA decay mechanisms in the host−pathogen interaction, Nonsense-mediated mRNA decay (NMD), and has revealed how viruses evade NMD and protect viral mRNAs [39,40,41,42]. These reports have shown that each virus has its own strategy to stabilize viral mRNAs; for example, by an inherent viral RNA stabilization mechanism, utilizing host RNA stability factors, inhibition of the host mRNA decay machinery, or hijacking the host cell RNA metabolism with viral nucleases [38,43].

**Figure 2:**
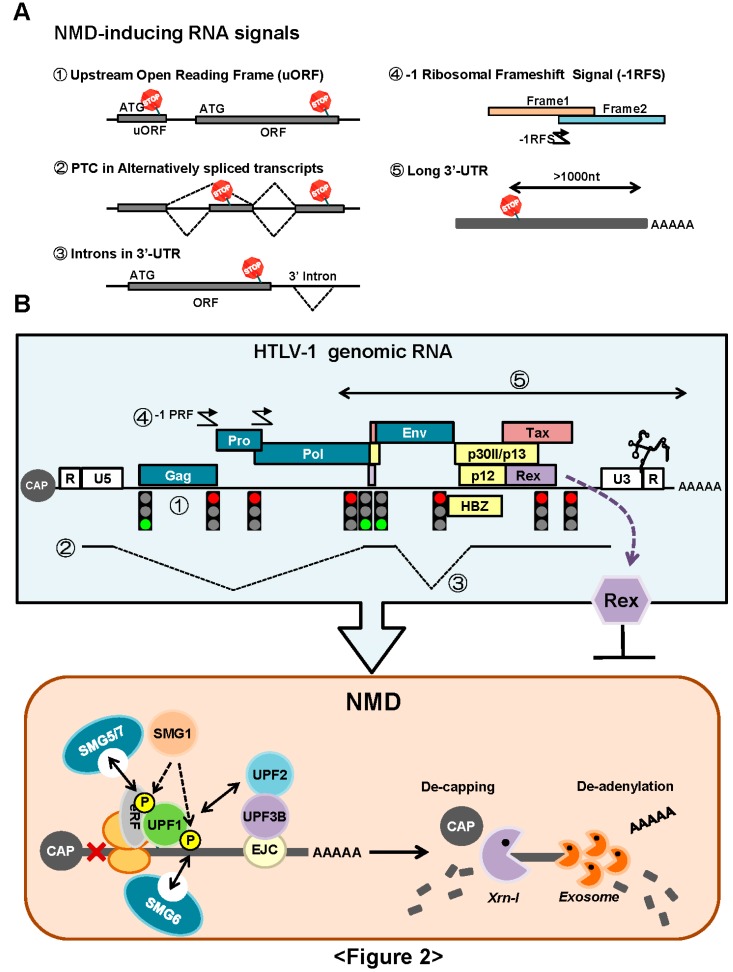
(**A**) NMD is an essential and evolutionarily-conserved cellular mRNA quality control mechanism. The principal function of NMD is to prevent the expression of harmful truncated proteins by selective elimination of aberrant mRNAs containing premature termination codons (PTCs). It has been reported that NMD also regulates the expression levels of normal mRNAs, which inherently contain RNA signals to generate PTC: 1. Upstream (U)-ORF, 2. alternative splicing producing PTC, 3. intron in 3′-UTR, 4. -1 programmed ribosomal frameshift signal (-1PRF) and 5. long 3′-UTR more than 1000 nt; (**B**) The genomic RNA of HTLV-1 contains various RNA signals potentially initiate NMD, *i.e.*, 1. multiple start and stop codons, 2. two alternative splicing sites, 3. intron in the 3′-UTR region of *gag/pro/pol* mRNA, 4. overlapping ORFs, and -1 PRF and 5. a long 3′-UTR (>1000 nt). We reported that HTLV-1 genomic RNA was indeed destabilized by NMD, and HTLV-1 Rex had a new function to inhibit NMD [32].

NMD is an essential and evolutionarily-conserved cellular mRNA quality control mechanism. The principal function of NMD is to prevent the expression of harmful truncated proteins by selective elimination of aberrant mRNAs containing premature termination codons (PTCs) (see reviews [43,44]) (Figure 2A). As indicated above, the major Rex function is the stabilization and export of the viral unspliced and partially-spliced mRNAs to cytoplasm. However, unspliced HTLV-1 mRNA (*i.e.*, viral genomic RNA) contains various RNA signals, such as multiple start and stop codons, overlapping ORFs, programmed ribosomal frameshift signals, and a long 3′-untranslated region (>1000 nt). These RNA signals are unusual for eukaryotic cells and have the potential to initiate NMD (Figure 2B). However, it is not clear how HTLV-1 evades NMD to protect its genomic RNA. Our laboratory has demonstrated that full-length HTLV-1 transcripts exhibit enhanced turnover in NMD-activated cells that overexpress UPF1, while knockdown of UPF1 by small interfering (si) RNA promotes enhanced stability of HTLV-1 genomic mRNA [45]. By confirming that the genomic and full-length mRNAs of HTLV-1 are sensitive to NMD, we further demonstrated that Rex inhibited NMD. We suggest that through the inhibition of NMD, Rex stabilizes viral transcripts in the cytoplasm to secure translation of viral structural proteins. In contrast, it is highly probable that Rex also perturbs cellular mRNA metabolism and host cell homeostasis by inhibition of the global NMD activity. It is noteworthy that Rex-mediated inhibition of NMD is not RNA- or sequence-specific, but Rex establishes a general blockage of NMD. Thus, not only the viral transcripts, but also natural host-encoded NMD substrates are stabilized in the presence of Rex. We demonstrated that Rex stabilized well-known NMD target mRNAs, such as *IL-6*, *MAP3K14*, and *FYN* mRNAs.

It has been reported that *IL-2Rα* mRNA was stabilized up to a five-fold level in Rex-overexpressing cells compared with the control cells without Rex [46,47], although the underlying mechanism has not been clarified. Since *IL-2Rα* mRNA can be a NMD target because of its upstream (u)-ORF structure, we speculate that this mRNA is stabilized by Rex through NMD inhibition. Indeed, UPF1 knockdown by siRNA in HeLa cells resulted in a significant increase in the *IL-2Rα* mRNA expression (Nakano unpublished data).

#### 3.1.2. How Does Rex Protect Viral mRNAs from NMD in the Cytoplasm?

NMD is a complex mechanism coupled with splicing and translation. Briefly, the core components of NMD are UPF1, UPF2, and UPF3, which detect the PTC-containing mRNA to be degraded via NMD, and SMG1, SMG5, SMG6, and SMG7, which phosphorylate/dephosphorylate UPF1 (Figure 2B). This results in the regulation of the activity of UPF1, the key molecule of NMD. UPF1 is a component of the termination complex, assembled when the ribosome reaches the termination codon of mRNA, while UPF2 and UPF3 are components of the exon junction complex (EJC) formed at the exon-exon boundary and are removed by the ribosome while it moves through. In normal mRNA, the termination codon is in the last exon, thus EJC does not remain at the end of translation. In contrast, PTC is located upstream of EJC, thus UPF1 on PTC comes into contact with UPF2 and UPF3, which triggers the phosphorylation of UPF1 by SMG1, and the onset of NMD. Phosphorylated UPF1 is dephosphorylated by the SMG5/SMG7 complex and recycled, while SMG7 completes the NMD process in mRNA processing bodies (p-bodies) of the cytoplasm. Recently, it was reported that SMG6 functioned as an endonuclease in the degradation of PTC-containing mRNA (see review [43]).

Since NMD machinery is coupled with splicing and translation apparatus, we speculate that Rex may influence the overall NMD activity directly via interaction with NMD core-components, and indirectly via interaction with splicing and translational machinery. Comprehensive protein-protein interactome analysis between Rex and the host-cellular proteins by a high-resolution mass spectrometry (MS) will be fruitful to understand the overall molecular landscape of NMD inhibition by Rex.

### 3.2. Regulation of mRNA Splicing Machinery by Rex

It is well known that mRNA splicing is coupled with transcription, and virtually all primary (unspliced) mRNAs are spliced at the site of transcription (see review [48]). Thus, HTLV-1 unspliced and partially-spliced mRNAs cannot evade splicing only through selective nuclear export by Rex. Consequently, we may speculate that Rex has a function to inhibit cellular splicing activity against the viral mRNAs, which is independent of the well-known CRM1-dependent nuclear export mechanism of Rex. Gröne *et al.* [49] demonstrated that Rex increased the nuclear quantity of unspliced viral mRNAs and reduced the number of spliced viral mRNAs. Since Rex did not influence the total quantity of viral transcripts, the authors conclude that Rex has a function by which it reduces splicing activity.

SF2/ASF regulates splicing activity and plays an important role in the splice-site selection [48,50]. Splicing patterns of HTLV-1 mRNA are governed by SF2/ASF *i.e.*, the differential pX splice site utilization of HTLV-1 mRNA is dependent on the expression level of SF2/ASF [51], although the viral mechanism to regulate the splicing activity through SF2/ASF has not been fully investigated. Interestingly, Powell *et al.* [52] demonstrated that HIV-1 Rev suppressed cellular splicing activity by recruiting SF2/ASF to the Rev-RRE RNP complex. Tange *et al.* [53] also showed the interaction between Rev and p32, the ASF/SF2-associated protein. The authors speculated that the interaction might function as a bridge between Rev and the host cellular splicing machinery. Considering the homologous function and molecular mechanism of HTLV-1 Rex and HIV-Rev, it is possible that Rex has a similar mechanism to suppress splicing machinery by binding and inhibiting the function of SF2/ASF.

Heterogeneous Nuclear Ribonucleoprotein A1 (hnRNPA1) is another cellular protein which is known to interact with Rex. hnRNPA1 associates with mRNA as a component of the RNP complex in the nucleus and influences the transcription, maturation and transport of mRNA [54]. HnRNPA1 also plays a crucial role in the regulation of alternative splicing, mainly as a splicing suppressor [48]. A recent study clearly showed that the expression level of hnRNPA1 had strong implications for the determination of exon-inclusion/skipping [55]. Hamaia *et al.* [56] first demonstrated that Rex function was impaired in a T cell line not infected by HTLV-1, Jurkat, and speculated that Rex was unable to bind to RxRE in the cell line. Later, the same study group found that hnRNPA1 bound to RxRE in competition with Rex, thus influencing the function of Rex [57]. Subsequently, Kress *et al.* [58] demonstrated that hnRNPA1 suppressed the Rex activity in a dose-dependent manner, while the suppression of hnRNPA1 in C91/PL, a HTLV-1-infected cell line, increased the Rex-dependent nuclear export of unspliced and partially-spliced mRNA. The authors proposed the possibility that hnRNPA1 enhances the splicing processes of viral mRNA. Indeed, hnRNPA1 caused enhanced exon 2 skipping in HTLV-1 mRNA [51]. On the other hand, the basal hnRNPA1 level was lower in HTLV-1-infected T cell lines (C91/PL, MT2, and HUT102) compared with other T cell lines without HTLV-1 infection (CBL and Jurkat) [57]. The authors concluded that HTLV-1 may have a mechanism to downregulate hnRNPA1, which is not advantageous for viral replication. Glutathione S-transferase (GST)-Rex pulldown assays conducted in our laboratory showed that Rex physically interacted with hnRNPA1 (Nakano, unpublished data). The underlying mechanism of how Rex is involved in the downregulation/inhibition of hnRNPA1 requires further investigation.

It has been shown that Rex changes the preference for exon usage during *FYN* mRNA splicing/maturation from exon7B to exon7A, resulting in enhanced production of the brain-type Fyn-B instead of the T cell-type Fyn-T [59]. Fyn is a proto-oncogene, belonging to the membrane-associated tyrosine kinase family. Its overexpression/disorder has been implicated to the tumorigenesis of several malignancies. Fyn has two major isoforms of distinct functions, Fyn-B expressed in the brain and Fyn-T expressed exclusively in hematopoietic cells, which are derived from exon7A and exon7B, respectively. Picard *et al.* [60] reported that the expression level of *FYN-B* mRNA was significantly increased in acute lymphoblastic leukemia or chronic lymphocytic leukemia. As indicated above, hnRNPA1, the regulator of exon usage, is downregulated in HTLV-1-infected cells. Moreover, we found that Rex interacts with hnRNPA1. If Rex itself is involved in the downregulation and/or suppression of hnRNPA1, such deregulation of hnRNPA1 function by Rex may have implications to alterations in the exon usage during mRNA maturation, such as observed in *FYN* mRNA. Aberrant overexpression of various splicing variants caused by genetic lesions in the splicing machinery may have implication to the HTLV-1 pathogenesis.

Taken together, the biological significance of the molecular interactions between Rex and the splicing-regulatory proteins in the regulation of splicing activity and splicing patterns requires elucidation in the future.

### 3.3. Cell-Cycle Regulation: Does Rex Interfere the Host Cell-Cycle Regulation?

A wealth of evidence has indicated that a number of viruses have mechanisms to modify cellular cell-cycle regulation for the promotion of viral replication. It has been well documented and reviewed that HIV-1 Vpr induces G2 arrest of the host cell-cycle [61,62,63]. The G2/M check point or DNA damage checkpoint is regulated by the activity of the Cdc2 (Cdk1) and CyclynB complexes, which are finely tuned by various kinases and phosphatases. Cdc2 undergoes inhibitory phosphorylation by Wee1 and Myt1, or Chk1/2, which are activated by the ATM/ATR DNA damage response pathway. Cdc25s are phosphatases and activate Cdc2 by dephosphorylation. When the cell senses DNA damage, Cdc2 is inhibited by the ATM/ATR pathway, and the cell cycle is arrested at G2. At the G2/M transition, PLK1 phosphorylates Cdc25s and Wee1 for activation and inhibition, respectively. Thus, Cdc2 is activated to enter the M phase (see review [64]). Furthermore, PLK1 is phosphorylated and activated by Aurora kinase A (AURKA) and its co-factor, Bora [65]. For the molecular mechanism of G2 arrest by Vpr, Zhao and Elder [61] indicated the importance of the interaction between Vpr and IκB kinase-associated serine/threonine protein phosphatase 2A (PP2A) in the induction of G2 arrest, although the detailed mechanism has yet to be clarified. Goh *et al.* [66] demonstrated that Vpr interacts with and inhibits Cdc25C. However, because Vpr is also known to activate ATR and Chk1 [67], it has not been fully elucidated whether Vpr directly inhibits Cdc25C or does so through the ATR pathway. Noronha *et al.* [68] investigated the influence of Vpr using a different methodology and showed that Vpr altered the subcellular localization of CyclinB1, Wee1, and Cdc25C. These authors also found that Vpr-induced herniations of the nuclear envelope and speculated that such disrupted nuclear architecture might interrupt normal cell-cycle progression.

**Figure 3:**
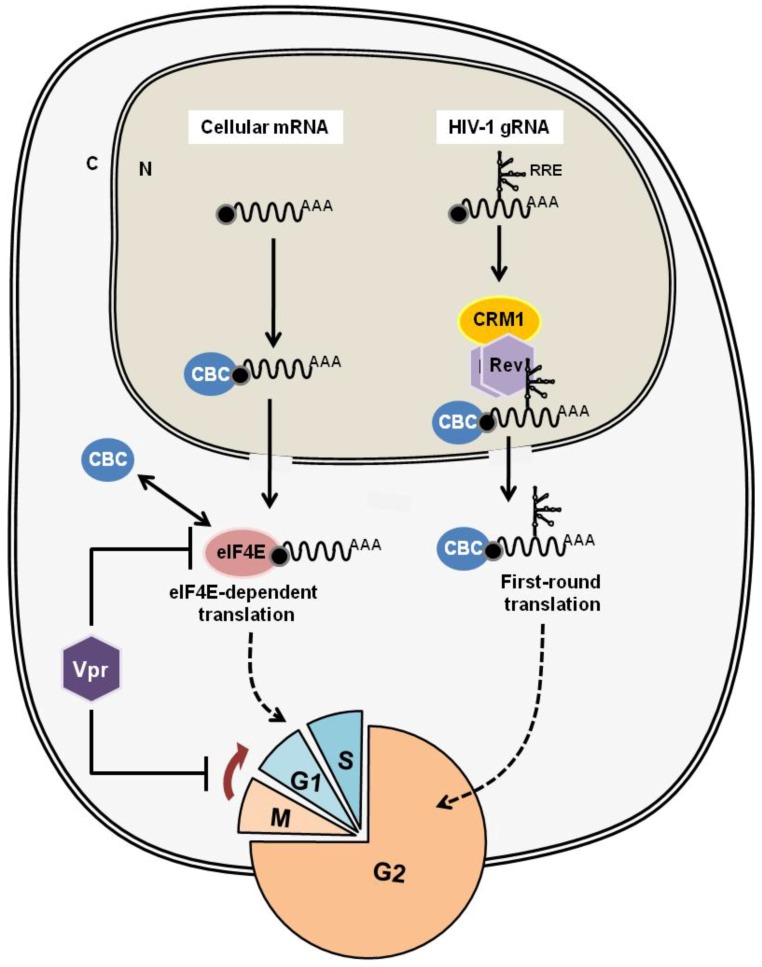
The G2-arrest by Vpr is beneficial for the selective translation of viral proteins. In the regular mRNA translation, the m^7^G-Cap structure of transcribed mRNA is first recognized by the Cap binding complex (CBC) and subjected to the pioneer round of translation for the quality check of mRNA. Then, CBC is replaced by the eIF4F complex for the steady-state translational procedure, which is regulated by eIF4E within the eIF4F complex. The majority of eIF4E-dependent translation is inhibited during G2 phase. On the other hand, CBC-dependent pioneer-round translation is cell-cycle independent. Since the translation of a viral protein from HIV-1 mRNA relies on CBC-dependent pioneer-round translation, G2-arrest by Vpr can enhance the translation of viral proteins by suppressing the eIF4E-dependent translation. Additionally, Vpr reduces the activated (phosphorylated) eIF4E level. Taken together, Vpr assists selective translation of HIV-1 mRNAs by induction of G2-arrest, as well as by suppression of eIF4E activity.

Why does HIV-1 Vpr induce G2 arrest? For viral replication, transcription from the provirus and translation of viral proteins are dependent on the host machinery. It is thought that G2 arrest by Vpr is beneficial for the selective translation of viral proteins (Figure 3). The m^7^G-Cap structure of transcribed mRNA is first recognized by the Cap binding complex (CBC) and subjected to the pioneer round of translation for the quality check of mRNA. Then, CBC is replaced by the eIF4F complex for the steady-state translational procedure, which is regulated by eIF4E within the eIF4F complex. It is thought that the translation of a viral protein from HIV-1 mRNA relies on CBC-dependent pioneer-round translation, which is cell-cycle independent. In contrast, the major eIF4E-dependent translation is inhibited during G2 phase. Therefore, G2-arrest by Vpr can enhance the translation of viral proteins (see review [69]). Furthermore, Sharma *et al.* [70] elegantly demonstrated that Vpr abrogates activated (phosphorylated) eIF4E levels. They also showed that CBC was retained at the Cap structure of unspliced and partially-spliced HIV-1 mRNAs in the cytoplasm. Taken together, these findings indicate that Vpr suppresses eIF4E activity by the reduction of its active form, as well as by the induction of G2-arrest. Thus, only CBC-bound HIV-1 mRNAs can be effectively subjected to cellular translational machinery (Figure 3). Most recently, it has been demonstrated that Vpr interacts with and activates the SLX4 endonuclease complex, which activates the DNA damage/repair response through the ATR/Chk1 pathway, resulting in G2 arrest [71].

HTLV-1, with a similar life-cycle to HIV-1, may have a similar strategy to enhance self-reproduction. However, the HIV-1 Vpr homologue has not been identified among the HTLV-1-encoded proteins. In the review by Zhao and Elder [61], they mention that HTLV-1 Tax showed similar characteristics to HIV-1 Vpr, such as binding to PP2A and the induction G2 arrest. Haoudi *et al.* [72] first indicated that Tax bound to and activated Chk2 in the DNA damage response, resulting in G2 arrest. Moreover, the interaction between Tax and Chk2 was further investigated by the same group, and they subsequently concluded that Tax inhibits the Chk2-induced DNA damage response through its retention in chromatin in order to evade the cellular DNA damage response to Tax-induced DNA instability [73]. Another study group also demonstrated that Tax bound to and inhibited the activity of Chk1, which is also involved in the ATM/ATR-mediated DNA damage response [74]. Fu *et al.* [75] showed that Tax interacted with PP2A to activate I kappa B kinase (IKK), thus influencing the nuclear factor (NF)-κB pathway. Together, these previous reports indicate that Tax inhibits the ATM/ATR-dependent DNA damage response and, thus, is not likely to induce G2 arrest. Anupam *et al.* [76] conducted a protein-interactome analysis for p30II and demonstrated that p30II interacted with ATM and modulated the activity of the G2/M checkpoint. There have been no reports implicating Rex to the cell-cycle regulation. Yet, we observed G2 arrest in CEM (ALL patient-derived human T cell line) overexpressing Rex (Nakano, unpublished data). Rex has arginine-rich NLS at the N-terminus similar to Vpr-NLS2, which is essential for G2 arrest. It has been demonstrated that eIF4E specifically binds to the mRNAs of cell-cycle promoting proteins in nucleus and is exported by CRM1. This mechanism is separated from TAP/NXF1 and REF/Aly-dependent export of bulk mRNAs [77,78,79,80]. Since Rex is also nuclear-exported by CRM1, we speculate that Rex may compete for CRM1 with eIF4E. Consequently, Rex may suppress the eIF4E-CRM1-dependent nuclear export of mRNAs encoding cell-cycle promoting proteins and, therefore, may induce cell-cycle arrest. The interaction between Rex and eIF4E and other cell-cycle regulating proteins should be investigated in the future.

## 4. Function of Rex and the Viral Pathogenesis

### 4.1. Do Rex-1/Rex-2 Functions Relate to the Pathogenesities of HTLV-1/HTLV-2?

Comparative analysis between Rex-1 (Rex) from HTLV-1 and Rex-2 from HTLV-2 can be helpful to understand the relationship between the function of Rex and the viral pathogenesity [13]. Both HTLV-1 and HTLV-2 belong to the same genus [81] and infect human T cells. Both viruses encode a similar set of viral proteins, including Tax and Rex and, thus, reproduce through a similar pathway. Yet, only HTLV-1 causes ATL and HAM/TSP in infected T cells, but not HTLV-2. The primary structures of Rex-1 and Rex-2 show 60% homology with common functional domains, such as RNA binding domain (RBD)/NLS, two multimerization domains, nuclear export signal (NES), and stability domain (SD) [24,25,26] and, thus, function as the viral RNA binding/transporting proteins through the common cellular pathways. On the other hand, the position of RxRE in the viral mRNA is different between HTLV-1 and HTLV-2, which may modulate impacts of Rex-1 and Rex-2 functions in their respective viral life cycles [13]. It has been clarified that all HTLV-1 mRNAs have RxRE , which is located in the U3/R region, while only unspliced HTLV-2 mRNA has RxRE, which is located in the R/U5 region [82]. Thus, it can be speculated that Rex-1 nuclear-exports all HTLV-1 mRNAs, including *tax/rex* mRNA, which enhances Tax/Rex expression and, thus, viral reproduction, whereas Rex-2 does not, resulting in a low viral production. Indeed, Bai *et al.* demonstrated that the nuclear export of the doubly spliced *tax/rex* mRNA of HTLV-1 was also enhanced by Rex-1 in a RxRE-1/CRM1-dependent manner [17]. Differences in nuclear export efficiencies of viral mRNA/Rex/CRM1 complex between HTLV-1 and HTLV-2 may influence viral replications and activities and, thus on pathogenesities of these viruses in infected T cells.

### 4.2. HTLV-1 Rex and HIV-1 Rev: Are They Similar or Different?

#### 4.2.1. HIV-1 Rev, the Molecular Counterpart of HTLV-1 Rex

**Figure 4:**
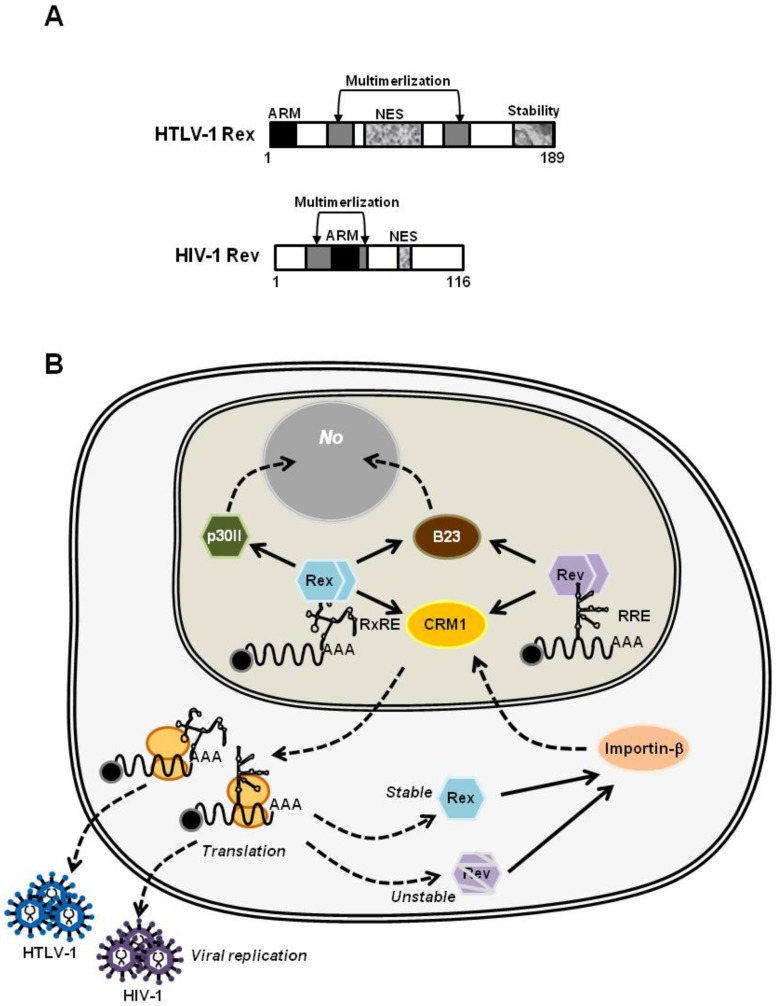
(**A**) The primary structures of HTLV-1 Rex and HIV-1 Rev. The homology in the primary sequences of HTLV-1 Rex and HIV-1 Rev is low, but they share most of the functional domains critical for their function, *i.e.*, arginine-rich RNA binding domain, NLS, NES, and two multimerization domains; (**B**) HTLV-1 Rex and HIV-1 Rev play similar functions through similar mechanisms. In the nucleolus, Rex and Rev specifically bind to the respective viral mRNAs through Rex responsive element (RxRE) for Rex and the Rev responsive element (RRE) for Rev. They stabilize unspliced or partially-spliced viral mRNA and actively transport these to the cytoplasm for selective translation of viral structural proteins by CRM1 binding through their NES. Rex and Rev return to the nucleus by binding to Importin-β, and further translocated to the nucleolus (No) by binding to B-23 via NLS.

Rev protein of HIV-1 (Human Immunodeficiency Virus type-I) is the molecular counterpart of HTLV-1 Rex. HTLV-1 and HIV-1 both belong to the family of *Retroviridae*, and are further specified to the genuses of *Deltaretroviru*s and *Lentivirus*, respectively. In addition, the major tropism of both viruses is human CD4^+^ T cells. HTLV-1 and HIV-1 have genomic RNA of a similar size, *i.e.*, about 8.5 knt and 9.75 knt, respectively, which encodes viral proteins with considerably homologous functions. HTLV-1 Rex and HIV-1 Rev bind viral mRNAs and shuttle between the nucleus and cytoplasm for nuclear export of viral transcripts, through quite similar mechanisms yet, interestingly, the homology between their amino acid sequences is very low [22,83]. Messenger-RNAs of HTLV-1 and HIV-1 have regions to form complex secondary structures called RxRE and RRE. Rex and Rev bind to viral mRNAs thorough RxRE and RRE as highly-specific landmarks, respectively. In terms of primary functional domains, both Rex and Rev have arginine-rich RNA binding domains for selective binding to their respective responsive elements (Figure 4A). It has been well documented that Rex and Rev stabilize unspliced and partially-spliced viral mRNAs, encoding viral structural proteins, and actively transport them to the cytoplasm for selective translation (Figure 4B). For shuttling between nucleus and cytoplasm, both proteins have NLSs for binding to importin-β and NESs for binding to CRM1 [23,83]. Furthermore, they bind to B-23 via NLSs to be translocated to the nucleolus [84,85,86,87] (Figure 4B). While the uniform role and mechanism of Rex and Rev are extensively discussed, some differences in the detailed molecular mechanism between Rex and Rev have been also described. It is now accepted that multimerization is essential for Rex to interact with RxRE, yet the monomer Rex is still able to bind to CRM1 for translocation to the cytoplasm [20,21,22]. Quite the opposite, the monomer Rev is known to bind to RRE, however multimerization of Rev up to 12 molecules is necessary for stable binding to CRM1 and for effective cytoplasmic-translocation [88,89]. These differences between these two viral RNA binding proteins may be closely related to the nuclear export efficiency of viral mRNAs, thus, viral replication and, consequently, to different disease associations, *i.e.*, ATL and HAM/TSP with HTLV-1, and AIDS with HIV-1.

#### 4.2.2. The Structural Biology of HTLV-1 Rex; Learning from that of HIV-1 Rev

Along with accumulation of knowledge in the molecular characteristics and functions, both Rex and Rev propose a common question; how these small viral proteins bind to RxRE/RRE of viral mRNAs and CRM1 simultaneously, and how they form a large, but stable, RNP complex for the nuclear export of viral mRNAs. To answer these questions, information from the structural biology may contribute significantly. In terms of structural biology, investigations into Rev have progressed far more than those of Rex. In contrast, our knowledge of the structure of Rex has not been updated from that of the N-terminal arginine-rich domain (aa 1–16) of the Rex peptide solved by nuclear magnetic resonance [90]. On the contrary, great efforts have been made to obtain the structural information on the Rev−Rev dimer interface or the Rev−RRE interaction since the early 2000’s. Daugherty *et al.* [91] clarified for the first time that Rev formed a homo-oligomer on the RRE via an oligomerization domain. In 2010, two different groups reported the partial structure of Rev in a dimer form [92,93]. Daugherty *et al.* [93] proposed the “jellyfish model” in which a rigid Rev dimer(s) forms an oligomeric structure, similar to the head of a jelly fish, while an unstructured NES region extended from each Rev molecule to form a tentacle-like alignment in binding to CRM1. They also mentioned that at the RNA-binding surfaces, “the two ARMs are arranged to reach out from the body of the Rev dimer to grasp RNA, much as two human arms are positioned to grip objects”.

Based on the Rev structure, the detailed molecular mechanism of HIV-1 viral mRNA nuclear export by Rev has since been clarified greatly. Fang *et al.* [94] investigated the structure of RRE thoroughly and demonstrated that RRE functioned as the topological landmark for Rev by forming an unusual structure, to which only Rev was able to specifically bind with high affinity. Finally, it was demonstrated that the RRE-Rev-CRM1 “HIV-1 export complex” was assembled co-transcriptionally at the transcription site, thus unspliced HIV-1 mRNAs were stably exported from the nucleus [95]. Moreover, it has been demonstrated that RRE-Rev assembly starts with changes in the RRE structure so that binding to the first two dimer complexes of Rev leads to further conformational changes of RRE, triggering oligomerization of Rev [96]. This enables stable binding to the CRM1 dimer [97]. Structural analysis of the Rev-dimer and RRE complex revealed that the Rev-dimer architecture can be flexibly altered depending on the structure of RRE [98]. The authors speculated that such changes in RRE structure and in the Rev-dimer architecture might alter the whole architecture of the “jellyfish complex” *i.e.*, the RRE-Rev-CRM1 complex. Overall, the structural biology of Rev indeed provided tremendous information to clarify the regulatory mechanism of the nuclear export efficiency of the HIV-1 export complex, thus resulting in pathogenesis of HIV-1. At present, the same questions still remain for HTLV-1 Rex. The structural biology of Rex is promising to provide significant information to answer these questions in the future.

## 5. Conclusions

**Figure 5:**
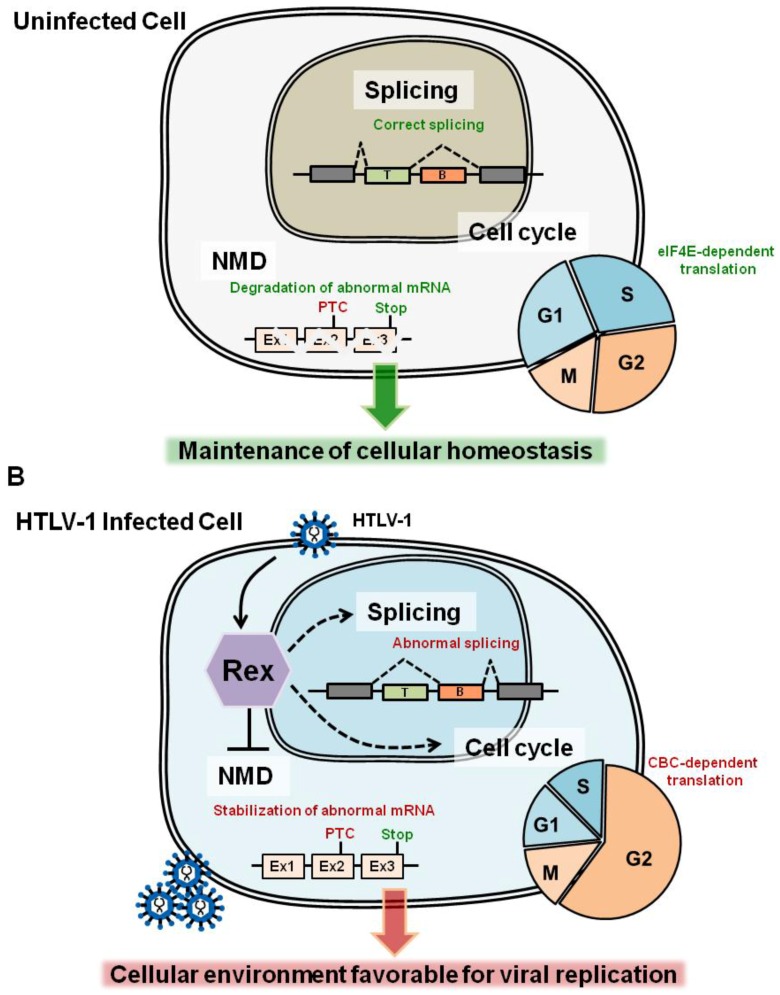
In this review, we focused on three cellular pathways, NMD, splicing machinery, and cell-cycle regulation, since we may expect that Rex interacts with these pathways to adjust the cellular environment suitable for the viral replication. (**A**) In the normal cells, the activities of NMD, splicing, and cell-cycle regulation are optimized to maintain the cellular homeostasis by eliminating PTC-containing harmful mRNAs, production of correctly spliced mRNAs encoding functional proteins, and adjusting the cell-cycle for n optimal cell proliferation rate and for effective eIF4E-dependent RNA translations, respectively; (**B**) In HTLV-1 infected cells, Rex inhibits NMD for stabilization of the viral genomic mRNA [32]. Additionally, based on previous reports and newly discovered aspects of Rex in our laboratory, we assume that Rex may suppress the activity of splicing machinery and may induce G2 arrest. These adjustments of host-cell mechanisms are favorable for the stabilization and translocation of viral mRNAs, as well as for selective translation of viral proteins for effective self-replication. On the other hand, suppression/alteration in these pathways may cause accumulation of abnormal PTC-containing mRNAs, thus harmful proteins; abnormal splicing patterns; deregulated cell proliferation; and suppression of eIF4E-dependent translation. Therefore, Rex-oriented tuning of the host cell environment may alter cellular homeostasis, and provide a basis for the pathogenesis of HTLV-1.

Accumulating data on the analysis of the Rex interactome shows that Rex has a significantly high potential to interact with a wide variety of cellular proteins. These cellular proteins are crucial for the maintenance of the cellular homeostasis by playing essential roles in mRNA surveillance and metabolism, nucleo-cytoplasmic shuttling, tumor growth regulation and in post-translational modification of proteins, such as SUMOylation [13,99,100]. These data strongly suggest that Rex modifies a wide range of cellular pathways in order to organize the host cellular environment suitable for the stabilization and translocation of viral mRNAs, as well as for selective translation of viral proteins for effective self-replication (Figure 5A). Such Rex-oriented tuning of the host cell environment can alter cellular homeostasis, and thus may provide a basis for the pathogenesis of HTLV-1 (Figure 5B).
